# Current perspectives on circadian regulation of mitochondrial dynamics in mood disorders and perioperative stress

**DOI:** 10.3389/fphar.2026.1723748

**Published:** 2026-02-26

**Authors:** Florencia Verbal, Nicole Rubilar, Ana M Marileo, Humberto Fierro, Oscar Guillermo Ramirez-Molina, Araceli Pinto-Leon, Gonzalo E Yevénes, Jorge Fuentealba, Jessica Panes-Fernández

**Affiliations:** 1 Mitochondrial Interactions in Neurological Disorders, Department of Physiology, Faculty of Biological Sciences, University of Concepción, Concepción, Chile; 2 Laboratory for the Screening of Neuroactive Compounds, Department of Physiology, Faculty of Biological Sciences, University of Concepción, Concepción, Chile; 3 Laboratory of Neuropharmacology, Department of Physiology, Faculty of Biological Sciences, University of Concepción, Concepción, Chile; 4 Center for Advanced Research in Biomedicine, University of Concepción, Concepción, Chile

**Keywords:** mitochondrial dynamics, circadian cycle, oxidative phosphorylation, mood disorders, perioperative stress, mitochondria, neurotransmission

## Abstract

Mitochondria act as a central integrative hub for oxidative phosphorylation, calcium homeostasis and metabolic signaling, reflecting their evolutionary origin from an α-proteobacterial endosymbiont. Although nearly 90% of their ancestral genes have been transferred to the nuclear genome, their role extends far beyond energy production. Emerging evidence positions mitochondria as active modulators of stress responses, which we term the “Mito-Mood Hypothesis.” This framework proposes that mitochondrial dynamics actively regulate gene expression and signaling, thereby shaping vulnerability to mood disorders such as depression, dysthymia, and seasonal affective disorder. Consistent with this view, patients with major depressive disorders show altered expression of nuclear-encoded mitochondrial genes, linking bioenergetics directly to psychiatric risk. We further discuss how oxidative phosphorylation (OXPHOS) modulates neurotransmitter cycles and how mitohormesis—adaptive responses to mild mitochondrial stress—can enhance resilience and cognition. Beyond psychiatry, mitochondrial vulnerability manifests in clinical settings: patients with mitochondrial diseases face elevated anesthetic risk, where agents such as propofol or volatile anesthetics may precipitate life-threatening metabolic crises. Collectively, these insights underscore mitochondria as central regulators of human health and highlight novel therapeutic opportunities bridging mood disorders and perioperative medicine.

## 1 Introduction

Mood disorders such as bipolar disorder, anxiety, and depression remain a major global public health burden, affecting millions across the world. In addition to their high prevalence, substantial treatment gaps persist, as shown in international surveys (Liu et al., 2024; Evans-Lacko et al., 2018). While their origins are complex and influenced by an interplay of genetic predisposition, environmental stressors, and neurochemical imbalances, recent findings point to a crucial and often underappreciated factor: Mitochondria behavior. These organelles—well recognized for their central role in ATP generation via oxidative phosphorylation—are increasingly recognized as active regulators of neuronal function (Clemente-Suárez et al., 2023).

Over the last two decades, several studies have demonstrated that mitochondrial dysfunction correlates with the pathophysiology of mood disorders. The association between mitochondrial DNA alterations and depression has been linked to impaired energy production, disrupted redox balance, and dysregulated calcium homeostasis. Brain imaging studies have shown that emotionally salient stimuli can induce changes in mitochondrial membrane potential in stress-responsive brain regions, even without an increase in energy demand (Jiang et al., 2024). This suggests that mitochondria play signaling roles beyond ATP production. In mood disorders such as major depression and bipolar disorder, mitochondrial alterations have consistently been documented, especially in the prefrontal cortex and hippocampus. These alterations include impaired respiratory complex activity, elevated oxidative stress, and changes in mitochondrial DNA (mtDNA) copy number, both in patients and in animal models (Scaini et al., 2021; Giménez-Palomo et al., 2021). Alongside this, we discussed how mitochondrial diseases resulting from mutations in either nuclear DNA (nDNA) or mtDNA may pose significant risks for patients undergoing anesthesia during surgical procedures, highlighting the importance of preoperative genetic and metabolic screening in individuals with potential mitochondrial disorders (Table 1). Table 1 outlines mood disorder–related vulnerabilities and their associated mitochondrial changes from a metabolic perspective. Here, we introduce the term ‘Mito-Mood Hypothesis’ to provide an integrative conceptual framework that proposes that specific mitochondrial alterations—such as oxidative phosphorylation, redox balance, and organelle dynamics—contribute to vulnerability to mood disorders by affecting neural circuit function and stress responsiveness. Box 1 provides a structured overview of this hypothesis.

Box 1 **The Mito-Mood Hypothesis: Conceptual Framework.**

**Conceptual Framework:** Mitochondria integrate energy production, redox balance, calcium signaling, and circadian cues to influence neuronal excitability and mood regulation. Unlike prior models, this hypothesis emphasizes dynamic mitochondrial responses to stressors, linking molecular, cellular, and circuit-level mechanisms.

**Association:** Mitochondrial changes—such as reduced OXPHOS efficiency, altered mtDNA integrity, and disrupted mitochondrial dynamics—are consistently reported in patients with mood disorders and correlate with behavioral phenotypes.

**Causation:** Experimental manipulations of mitochondrial function can induce or reverse mood-related phenotypes in preclinical models.

**Testable Predictions:** Mitochondrial markers (mtDNA, OXPHOS proteins, NAD^+^/NADH) reflect mood severity and treatment response. Changes in mitochondrial dynamics in limbic regions alter neural circuits.

Stabilizing mitochondrial function pharmacologically or via behavioral interventions improves mood and resilience.

1. Circadian modulation of mitochondrial output predicts diurnal mood fluctuations.

**Summary:** Mitochondria act as dynamic hubs linking metabolism, neuronal activity, and affective regulation, providing a mechanistic scaffold for experimental and translational studies.

**TABLE 1:** Mitochondrial dysfunction is associated with mood disorder symptoms.

Mood disorder symptom or vulnerability	Mitochondrial change (metabolic perspective)	References
Chronic stress vulnerability/stress-induced depression	↑ Oxidative stress, mtDNA damage, ↓ energy production, ↑ membrane permeability, ↑ pro-apoptotic signaling (mitochondrial allostatic load)	Allen et al. (2021), Liu et al. (2025)
MDD	↓ Mitochondrial quality control, ↓ biogenesis, altered dynamics (fusion/fission/mitophagy), impaired ETC function, ↑ ROS production	Song et al. (2023), Chen et al. (2024), Jiang et al. (2024)
Social chronic stress → depressive behaviors	↑ Neuroinflammation and ↑ ROS production	Hollis et al. (2022), Liu et al. (2025)
Depression and plasticity/resilience	Altered intracellular and mitochondrial Ca^2+^ signaling; dysfunctional Ca^2+^ buffering and uptake	Song et al. (2023), Khan et al. (2023)
Bipolar disorder – depressive phase	↓ Respiratory capacity, ↓ activity of ETC Complexes I–IV (reversible during remission)	Chen et al. (2024), Lam et al. (2023)
Bipolar disorder – manic phase	↓ Respiratory capacity, ↓ activity of ETC Complexes I–IV (*changes reported as reversible during remission*)	Kageyama et al. (2025), Chen et al. (2024)

MDD, major depressive disorder; OXPHOS, oxidative phosphorylation; ROS, reactive oxygen species; mtDNA, mitochondrial DNA; ETC, electron transport chain; Ca^2+^, calcium; PGC-1α, peroxisome proliferator-activated receptor gamma coactivator 1-alpha; TFAM, mitochondrial transcription factor A; MAO-A, monoamine oxidase A.

## 2 Global burden of mood disorders

Mood disorders represent one of the leading contributors to the global burden of disease, with lifetime prevalence estimates ranging from 10% to 20% across populations (Chan et al., 2024). According to the World Health Organization (WHO), more than 280 million people are affected by depression worldwide, while anxiety disorders impact over 300 million individuals, and approximately 40 million people live with bipolar disorder (Chan et al., 2024; Chodavadia et al., 2023; Nierenberg et al., 2023; Berk et al., 2025). Together, these conditions account for nearly 15% of global years lived with disability (YLDs) and rank among the top five causes of disability-adjusted life years (DALYs) in young and middle-aged adults. Beyond their high prevalence, mood disorders carry substantial economic and social costs, including reduced productivity, increased healthcare utilization, and elevated risk of suicide, which claims nearly 700,000 lives each year (Lovero et al., 2023). Despite the availability of pharmacological and psychotherapeutic interventions, treatment gaps remain considerable: up to 50% of individuals in high-income countries and over 80% in low- and middle-income countries receive no adequate treatment (Thornicroft et al., 2017). Moreover, current pharmacological strategies face major limitations: first-line antidepressants achieve remission in only 30%–40% of patients, often requiring prolonged treatment trials and combination strategies (Rush et al., 2006). Delayed onset of action, high relapse rates, and limited efficacy for bipolar depression further highlight the unmet clinical need. Gender differences also shape disease vulnerability and outcomes: women are nearly twice as likely as men to develop major depressive disorder, exhibit greater symptom burden, and face higher relapse risk, particularly during reproductive transitions such as postpartum and menopause (Tseng et al., 2023; Radoš et al., 2024). In Chile, epidemiological surveys reveal comparable or even higher burdens: the National Health Survey (2016–2017) estimated that 6.2% of Chileans over 15 years old meet clinical criteria for depression, while nearly 23.6% of adults present some form of mental disorder (Brody, 2022). Recent epidemiological analyses in Chile have shown that depressive symptoms are highly prevalent among adults, affecting nearly one in eight individuals, consistent with findings from the National Health Surveys indicating substantial mental health burdens across the population (Luna et al., 2024). In Chilean adolescents, the prevalence of depressive and stress-related symptoms increased markedly during and after the COVID-19 pandemic. Recent nationwide data indicate that up to 60% of students report depressive symptoms and more than 50% experience significant stress levels, reflecting a major mental health burden. Moreover, suicidal ideation reached approximately 10%–11%, with female adolescents being disproportionately affected (Bustos Villarroel et al., 2024). These epidemiological realities underscore the urgent need for innovative conceptual frameworks and therapeutic strategies that address the biological underpinnings of mood disorders.

## 3 Mitochondria and circadian genes shape brain energy and emotional stability

Neurons are the most energy-demanding cells in the human body, reflecting their wide range of biochemical activities (Table 2). Table 2 summarizes biophysical estimates of ATP consumption by major energy-dependent neuronal processes, highlighting that maintenance of ionic gradients, calcium handling, and synaptic transmission together account for the majority of neuronal energy demand and place mitochondria at the center of synaptic homeostasis. Through OXPHOS, mitochondria sustain the high metabolic needs of neurons, supporting synaptic transmission, maintenance of ionic gradients, and axonal transport (Howarth et al., 2012; Attwell and Laughlin, 2001). Mitochondria finely regulate intracellular calcium dynamics, acting as buffers that modulate local calcium transients critical for neurotransmitter release and synaptic plasticity (Giménez-Palomo et al., 2021). Disruptions in this calcium-handling capacity can trigger excitotoxic cascades, contributing to synaptic dysfunction and mood instability (Giménez-Palomo et al., 2021). The dynamic nature of mitochondria—continuously undergoing fission and fusion—ensures their distribution along complex neuronal arbors and facilitates mitophagy, the selective clearance of damaged mitochondria (Chen et al., 2024). Perturbations in these dynamics, involving proteins like DRP1, OPA1, PGC-1α, and MFN2, have been associated with cognitive impairments and mood disorders (Scaini et al., 2021; Scaini et al., 2022; Ulecia-Morón et al., 2025). Moreover, mitochondria govern intrinsic apoptotic pathways, releasing pro-apoptotic factors such as cytochrome c in response to stress, a process that, when dysregulated, contributes to the selective neuronal loss seen in affective disorders (Song et al., 2023; Chen et al., 2024). Advances in live-cell imaging and molecular tracking now allow us to appreciate that mitochondrial function is not static but oscillates with circadian cycles, imparting a temporal “bioenergetic signature” that may underlie diurnal variations in neuronal excitability and mood (Schmitt et al., 2018; Neufeld-Cohen et al., 2016; Li et al., 2025). Peaks in ATP levels enhance action potential firing and neurotransmission, while rhythmic ROS fluctuations influence the activity of redox-sensitive ion channels, including NMDA receptors, thus coupling metabolic states to synaptic activity (Feofilaktova et al., 2025).

**TABLE 2:** Biophysical estimates of ATP consumption by energy-dependent neuronal processes per neuron or per synapse, with mitochondrial implications.

Process	ATP consumption (molecules/Neuron/s)	Function	Mitochondrial relevance	References
Na^+^/K^+^-ATPase	∼4.3 × 10^9^	Maintains membrane potential by exchanging 3 Na^+^/2 K^+^ ions	Primary ATP consumer; deficits impair excitability and synaptic signaling	Howarth et al. (2012)
SERCA and PMCA	∼1.2 × 10^8^	Clear cytosolic Ca^2+^ (SERCA to ER, PMCA to extracellular space)	ATP-dependent buffering; failure leads to synaptic dysfunction and excitotoxicity	Attwell and Laughlin (2001)
Neurotransmitter Recycling	∼3.0 × 10^8^	Reuptake, enzymatic degradation and vesicle reloading	Mitochondrial ATP-dependent transporters and vesicular refilling	Attwell and Laughlin (2001)
Vesicular Acidification	∼4.0 × 10^8^	V-ATPase loads protons into vesicles for neurotransmitter uptake	ATP-dependent; reduced acidification impairs synaptic transmission	Egashira et al. (2015)
Glycine Synthesis and Cycling	∼6.4 × 10^7^	Involves serine-glycine interconversion, GlyT1/2, and NMDA co-activation	Dependent on NADH and ATP; disruption affects inhibition and NMDA signaling	Attwell and Laughlin (2001)

ATP, adenosine triphosphate; Na^+^/K^+^ ATPase, sodium-potassium adenosine triphosphatase; SERCA, sarco/endoplasmic reticulum Ca^2+^-ATPase; PMCA, plasma membrane Ca^2+^-ATPase; Ca^2+^, calcium ion; ER, endoplasmic reticulum; NADH, nicotinamide adenine dinucleotide (reduced form); GlyT1/2, glycine transporter types 1 and 2; NMDA, N-methyl-D-aspartate receptor; V ATPase, vacuolar-type H^+^-ATPase.

**FIGURE 1:**
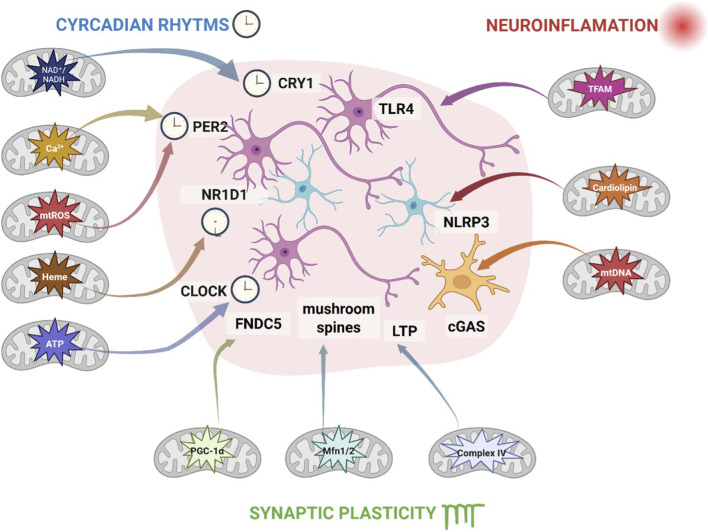
Mito-Mood Hypothesis. Mitochondria act as a central hub integrating circadian rhythms (blue), neuroinflammation (red), and synaptic plasticity/LTP (green). Circadian metabolites (NAD^+^/NADH, mtROS, heme, ATP) regulate clock proteins (CLOCK, PER2, CRY1, NR1D1) and FNDC5, promoting mushroom spine formation. Mitochondrial DAMPs (TFAM, cardiolipin, mtDNA) activate innate receptors in glia (TLR4, NLRP3, cGAS), driving pro-inflammatory cytokine release. Synaptic plasticity relies on mitochondrial ATP, Ca^2+^, ROS, and regulatory proteins (PGC-1α, Mfn1/2, Complex IV), supporting LTP and dendritic spine remodeling. Arrow colors indicate signaling type: blue/green for energy/plasticity, red for neuroinflammation.

These rhythmic changes in mitochondrial output likely interact with extracellular concentrations of monoamines, glutamate, GABA, and neuropeptides, forming a bidirectional regulatory interface between mitochondrial activity and neurotransmitter signaling (Ragozzino et al., 2023; Tanaka et al., 2022). Disruption of this neurochemical synchrony may play a key role in the pathophysiology of mood disorders (Tanaka et al., 2022). Taken together, mitochondria emerge not merely as passive suppliers of energy but as highly dynamic organelles whose morphology, positioning, and activity are critically involved in the stability of neural networks and the regulation of affective states. We therefore propose the ‘Mito-Mood Hypothesis’ as a synthesized framework in which mitochondria function as key integrators that transduce environmental stressors, circadian cues, neuroinflammatory signals, and metabolic states into changes in nuclear gene expression, redox tone, and synaptic function. This unifying perspective offers a mechanistically grounded theoretical model for understanding how mitochondrial dysfunction contributes to psychiatric risk and cognitive outcomes (Figure 1).

Preclinical and systems-level studies indicate that these circadian factors influence mitochondrial biogenesis, dynamics, and redox tone. During the day, the promoters CLOCK and BMAL1 are active, stimulating the transcription of PER and CRY genes, whereas at night, the accumulated PER/CRY proteins inhibit CLOCK/BMAL1 activity, silencing clock-controlled genes until their degradation resets the cycle: (i. (Jacobi et al., 2015)) *BMAL1* promotes mitochondrial biogenesis and respiratory capacity by modulating the expression of OXPHOS-related genes; (ii. (Chang and Guarente, 2013)) *CLOCK* interacts with *SIRT1* to influence mitochondrial transcription factors and fusion machinery; (iii. (Weng et al., 2021)) *PER2* loss is associated with altered ROS homeostasis and impaired mitophagy; (iv. (Jordan et al., 2017)) *CRY1/2* modulate mitochondrial redox responses and calcium buffering; and (v. (Zhang-Sun et al., 2023)) *NR1D1* (REV-ERBα) couples lipid metabolism with mitochondrial bioenergetics through repression of BMAL1 and control of mitochondrial turnover. These molecular checkpoints integrate circadian rhythmicity with the energetic and redox demands of neuronal activity, thereby providing a mechanistic link between clock gene dysregulation, mitochondrial dysfunction, and the emergence of mood-related phenotypes.

**FIGURE 2:**
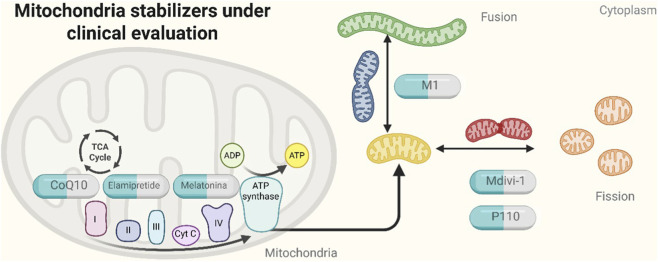
Mitochondria stabilizers under preclinical evaluation and their sites of action. Schematic representation of compounds currently undergoing preclinical evaluation for mitochondrial stabilization. CoQ10, coenzyme Q10; ETC, electron transport chain; ATP, adenosine triphosphate; DRP1, dynamin-related protein 1; Fis1, mitochondrial fission 1 protein; M1, *mitochondrial fusion promoter 1*; Mdivi-1, *mitochondrial division inhibitor 1*; TCA, *tricarboxylic acid* (cycle).

This conceptual framework highlights the therapeutic potential of targeting mitochondrial dynamics to restore neuroenergetic balance (Chen et al., 2024). As illustrated in Figure 2, several mitochondria-targeted compounds are under clinical evaluation for their capacity to stabilize mitochondrial function at multiple levels. Coenzyme Q10 has demonstrated efficacy in improving mitochondrial bioenergetics in Parkinson’s and mood disorders (Cleren et al., 2008; Mehrpooya et al., 2018), while Elamipretide (SS-31) is currently in phase II trials for mitochondrial myopathy and age-related disorders, with promising neuroprotective effects in preclinical models of depression (Koňaříková et al., 2020; Zhang et al., 2024a). Melatonin, beyond its role as a circadian modulator, directly stabilizes mitochondrial membranes and reduces ROS production, as well as showing antidepressant-like effects in both clinical and preclinical studies (Reiter et al., 2016; Li et al., 2024c). Compounds targeting mitochondrial dynamics, such as M1 (a fusion enhancer) and Mdivi-1 (a DRP1 inhibitor), have been shown to restore mitochondrial morphology and function in animal models of neurodegeneration and mood disorders (Nibrad et al., 2025; Grohm et al., 2012; Bordt et al., 2017). P110, a selective DRP1-Fis1 interaction blocker, prevents excessive mitochondrial fragmentation and has entered early-stage clinical testing in neurodegenerative diseases (Qi et al., 2013; Sridharan et al., 2024). Light therapy and behavioral training may also synergize with mitochondrial stabilizers to reestablish rhythmic bioenergetic profiles. By simultaneously targeting mitochondrial bioenergetics and circadian entrainment, such combinatorial approaches offer a promising avenue for reestablishing neuronal metabolic-electrical coupling—a core deficit in mood disorders (Wider et al., 2023; Arranz-Paraíso et al., 2023; Tong et al., 2024) (Table 3). To avoid overstating therapeutic implications, Table 3 summarizes mitochondria-targeted interventions according to their primary mitochondrial targets, level of evidence, reported outcomes, and key limitations, clearly distinguishing preclinical findings from clinical and perioperative data. Further clinical investigation is warranted to validate the translational potential of this dual-target model in bipolar disorder, major depressive disorder, and related affective syndromes.

**TABLE 3:** Evidence map of mitochondria-targeted interventions relevant to mood disorders and perioperative vulnerability.

Compound/Intervention	Primary mitochondrial target	Evidence level	Outcomes reported	Key limitations	References
Melatonin	Improves ETC efficiency, reduces ROS, stabilizes mtDNA	RCTs (mood disorders) + observational	Mild improvement in mood symptoms; reduced oxidative stress markers	Small sample sizes; heterogeneity of dosing; few perioperative studies	Reiter et al. (2016), Tiong et al. (2019), Reiter et al. (2017)
MitoQ (mitochondria-targeted antioxidant)	Reduces mitochondrial ROS, improves Complex I function	Preclinical + early-phase human studies	Decreases oxidative stress biomarkers; limited mood data	Lack of robust clinical trials; unclear perioperative impact	Smith and Murphy (2011), Snow et al. (2010), Rossman et al. (2018)
Elamipretide (SS-31 peptide)	Stabilizes cardiolipin and improves OXPHOS coupling	Phase 1–2 trials + strong preclinical	Improved mitochondrial function markers; no conclusive mood clinical endpoints	No RCTs in psychiatric or perioperative settings	Szeto and Birk (2014), Birk et al. (2013), Zhao et al. (2004), Pharaoh et al. (2023)
N-acetylcysteine (NAC)	Antioxidant; supports glutathione synthesis	Multiple RCTs in mood disorders	Some benefit in depressive symptoms; improved redox biomarkers	Mixed results; no perioperative RCTs	Berk et al. (2008), Fernandes et al. (2016), Deepmala et al. (2015)
α2-agonists (dexmedetomidine)	Reduces mitochondrial stress during anesthesia; increases metabolic efficiency	Clinical perioperative studies (non-mitochondrial primary endpoints)	Reduced postoperative delirium; better hemodynamic stability	Mechanistic mitochondrial data indirect; limited psychiatric data	Deng et al. (2024), Gertler et al. (2001), Brown et al. (2011), Su et al. (2016)
Ketogenic interventions	Enhances metabolic flexibility; increases NAD^+^/NADH	Preclinical + small human studies	Biomarker improvement; possible mood symptom benefit	Adherence and safety concerns; not tested perioperatively	Newman and Verdin (2014), Hasan-Olive et al. (2019), Ozan et al. (2024), Pietrzak et al. (2022)
Botanical flavonoids (e.g., quercetin)	Antioxidant, modulates Nrf2/AMPK	Preclinical studies	Reduced ROS; improved mitochondrial markers in animals	No clinical trials; uncertain dosing and purity	Li et al. (2016), Kicinska and Jarmuszkiewicz (2020), Dajas, 2012; Wang et al. (2021)

## 4 Functional alterations in mitochondrial pathways and their impact on mood disorders

**FIGURE 3:**
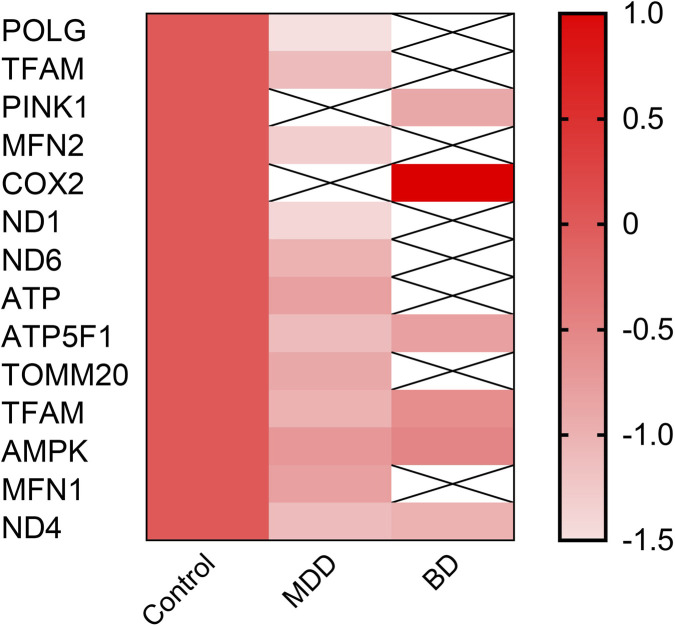
Heatmap summarizing key patterns of change in mitochondrial-related gene expression. Gene symbols displayed in the figure include validated mitochondrial-related genes (POLG, TFAM, PINK1, MFN2, COX2, ND1, ND4, ND6, and ATP5F1). Remaining labels correspond to source-specific dataset annotations. Red color intensity indicates increased values relative to control conditions. Values reflect qualitative directionality rather than effect size. This heatmap is an illustrative integrative summary and does not represent a quantitative meta-analysis, given the heterogeneity in sample types, experimental designs, and analytical methodologies.(Scaini et al., 2021; Wu et al., 2025; Kim et al., 2017; Li et al., 2024a; Çeli et al., 2023; Scaini et al., 2022; Ulecia-Morón et al., 2025; Kasahara et al., 2016; Gautier et al., 2008; Wang and Dwivedi, 2017; Ceylan et al., 2024; Scarr et al., 2019). Abbreviations: POLG, mitochondrial DNA polymerase gamma; TFAM, mitochondrial transcription factor A; PINK1, PTEN-induced kinase 1; MFN2, mitofusin-2; COX2, cytochrome c oxidase subunit 2; ND1/ND4/ND6, mitochondrial Complex I subunits; ATP5F1, ATP synthase F1 subunit.

Multiple lines of evidence suggest an association between mitochondrial-related genes and mood disorders; accordingly, Figure 3 is presented as an illustrative, qualitative summary rather than a quantitative meta-analysis, reflecting the heterogeneity of the underlying studies (Figure 3). Clinical imaging studies, such as magnetic resonance spectroscopy (MRS), have shown reduced levels of ATP and phosphocreatine in the brains of patients with late-onset MDD (Wu et al., 2025; Scaini et al., 2021). Decreases in the NAD^+^/NADH ratio indicate impaired redox balance, suggesting systemic energetic failure in the central nervous system (Cantó et al., 2015). These bioenergetic impairments are accompanied by increased oxidative stress and mtDNA damage (Kim et al., 2017; Li et al., 2024a). Elevated levels of 8-OHdG (8-hydroxy-2′-deoxyguanosine), a marker of oxidative DNA damage, have been reported in blood and cerebrospinal fluid of patients with mood disorders (Çeli et al., 2023). Experimental models of depression in rats have demonstrated reduced activity of mitochondrial respiratory chain complexes, particularly Complexes I and IV (Rezin et al., 2008). Rodent models subjected to chronic unpredictable stress (CUS) or early life stress display reductions in mitochondrial density, membrane potential, and respiratory capacity in the hippocampus and prefrontal cortex (Ulecia-Morón et al., 2025). These mitochondrial alterations have been suggested to be associated with behavioral phenotypes relevant to mood disorders, including anhedonia, social withdrawal, and learned helplessness, primarily in chronic stress–based animal models (Rezin et al., 2008; Morais et al., 2012; Ulecia-Morón et al., 2025). In Polg mutator mice, which accumulate mtDNA mutations due to impaired proofreading, animals show increased immobility in forced swim and tail suspension tests (Kasahara et al., 2016). Likewise, PINK1 and Parkin knockout models, originally developed for Parkinson’s disease, also display depressive-like behavior and altered mitochondrial dynamics, especially in limbic regions (Gautier et al., 2008). Transcriptomic profiling of postmortem prefrontal cortex from patients with Major Depressive Disorder (MDD) and Bipolar Disorder (BD) revealed altered expression of multiple mitochondria-associated genes compared with healthy controls, highlighting disruptions in pathways of biogenesis, OXPHOS, and mitophagy (Wang and Dwivedi, 2017). Epigenetic and transcriptomic studies suggest that mitochondrial biogenesis regulators, including POLG and TFAM, may be dysregulated in mood disorders, potentially contributing to impaired mtDNA maintenance and replication (Ceylan et al., 2024). Genes involved in OXPHOS have been reported to show altered expression in mood disorders, supporting dysfunction in electron transport chain (ETC) activity and ATP synthesis (Giménez-Palomo et al., 2021). Notably, transcripts linked to mitochondrial dynamics and quality control, such as *PINK1* and *MFN2*, also display significant changes, particularly reductions, supporting the hypothesis of defective mitophagy and disrupted fusion-fission balance in the brains of affected individuals (Scaini et al., 2022). These alterations are heterogeneous across patient samples; some show gene-specific downregulation or upregulation, and in certain cases, data may be missing or statistically non-significant, reflecting the heterogeneity of mood disorders at the molecular level (Scarr et al., 2019).

Pharmacological interventions targeting mitochondria further highlight the functional role of mitochondrial health in mood regulation. Chronic administration of mitochondrial toxins such as rotenone (a Complex I inhibitor (Morais et al., 2012)) or 3-nitropropionic acid (a Complex II inhibitor) induces depressive-like behaviors in rodents (Li et al., 2008). Conversely, compounds like N-acetylcysteine (NAC) (Deepmala et al., 2015), coenzyme Q10 (Aboul-Fotouh, 2013)—which support antioxidant defenses and ETC efficiency—have shown antidepressant-like effects in both animal models and preliminary clinical trials. For example, NAC modulates glutathione levels and redox signaling, and improves mitochondrial respiration in stressed rats, correlating with improved affective behavior (Kim et al., 2024). However, Randomized Controlled Trials (RCTs) in humans have yielded mixed results, with some studies reporting clinical benefit and others showing limited or no efficacy (Pandya et al., 2013).

Despite this growing body of evidence, critical gaps remain. First, causality is still difficult to establish—most findings are associative or based on indirect measures of mitochondrial function. Second, mitochondrial changes may result from chronic inflammation, glucocorticoid toxicity, or other upstream factors rather than constituting a primary cause. Third, the translational value of preclinical models is limited by species differences in mitochondrial dynamics and behavioral readouts. There is also a lack of validated biomarkers to stratify patients or monitor mitochondrial interventions in clinical trials. In this context, the field requires better longitudinal human studies, integrative multi-omics approaches, and improved targeted therapies that can cross the blood-brain barrier and engage specific mitochondrial pathways relevant to mood regulation.

## 5 Mitochondrial disease and anesthesia: clinical challenges and risk of fatal outcomes

Mitochondrial diseases (MDs) are a heterogeneous group of inherited disorders resulting from defects in mitochondrial OXPHOS (Gorman et al., 2016). These diseases can arise from mutations in either nuclear nDNA or mtDNA, resulting in impaired ATP production and increased reliance on anaerobic pathways (Schon et al., 2012). Tissues with high energy demands—such as the brain, skeletal muscle, heart, and liver—are particularly vulnerable (Wallace, 2005). Anesthesia poses a significant clinical challenge in patients with MDs due to their underlying metabolic vulnerability. The physiological stress of surgery, fasting, exposure to anesthetic agents, and temperature fluctuations can precipitate metabolic decompensation, lactic acidosis, respiratory failure, and even death (Footitt et al., 2008). Although many patients tolerate anesthesia uneventfully, multiple fatal outcomes have been reported in case reports and small series, underscoring the importance of tailored anesthetic management (Niezgoda and Morgan, 2013).

An impaired bioenergetic state is thought to reduce patients’ tolerance to additional metabolic stressor. This bioenergetic deficit is often linked to mutations in mtDNA or nDNA affecting key components of the OXPHOS system (Koopman et al., 2012).

Common mtDNA mutations include m.3243A>G (associated with MELAS syndrome), m.8344A>G (linked to MERRF) (Mancuso et al., 2014), and large-scale mtDNA deletions observed in Kearns-Sayre syndrome (KSS) (Grigalionienė et al., 2023). On the nuclear side, mutations in genes such as POLG (the mitochondrial DNA polymerase gamma) (Rajakulendran et al., 2016), SURF1 (linked to Leigh syndrome) (Lee and Chiang, 2021), and NDUFS1/NDUFS4 (Complex I subunits) (Dang et al., 2020), disrupt ETC assembly (Stumpf et al., 2013). These mutations have been associated with increased sensitivity to volatile anesthetics and a higher risk of anesthetic-related metabolic decompensation (Hsieh et al., 2017). Furthermore, patients with mitochondrial encephalomyopathies (e.g., MELAS, Leigh syndrome) often exhibit multi-organ involvement, including cardiomyopathies, seizures, respiratory muscle weakness, and renal tubular dysfunction, all of which influence anesthetic planning and risk stratification (Niezgoda and Morgan, 2013).

A comprehensive preoperative evaluation is essential for patients with mitochondrial disease, with particular attention to metabolic status (including lactate, glucose, bicarbonate, and pH), cardiac function (ECG and echocardiogram to assess for cardiomyopathy or conduction defects), respiratory capacity—especially in cases with neuromuscular involvement—and nutritional status, including any history of prior anesthetic complications (Niezgoda and Morgan, 2013; Kurnutala and Hubbard, 2023). Multidisciplinary consultation with neurology, genetics, and metabolic specialists is strongly recommended. Prolonged fasting should be avoided, and intravenous glucose may be administered preoperatively to prevent catabolic stress. In terms of anesthetic agents, propofol should be used with extreme caution due to its association with Propofol Infusion Syndrome (PRIS), a potentially fatal complication particularly dangerous in the context of mitochondrial dysfunction, especially with Complex I deficiency (Hsieh et al., 2017). Etomidate and ketamine may be safer alternatives, though the latter’s sympathomimetic effects warrant caution in patients with cardiomyopathy (Hsieh et al., 2017). Volatile anesthetics have been shown to inhibit mitochondrial Complexes I and II and have been associated with postoperative respiratory failure (Hsieh et al., 2017; Hsieh et al., 2021). Notably, a fatal case was reported in a child with Leigh syndrome, likely due to central respiratory suppression (Hsieh et al., 2017; Morgan et al., 2002). Non-depolarizing neuromuscular blocking agents require careful titration and monitoring, while succinylcholine is generally avoided due to the risk of hyperkalemia and rhabdomyolysis, particularly in myopathic phenotypes (Niezgoda and Morgan, 2013). Adjunct medications should emphasize opioid-sparing strategies—dexmedetomidine offers benefits due to minimal respiratory depression—and antiemetics like ondansetron and dexamethasone are generally well tolerated (Niezgoda and Morgan, 2013). Intraoperative management should prioritize the maintenance of normoglycemia with IV glucose, prevention of hypothermia using warming devices, and close monitoring of lactate and acid-base status through serial blood gases (Parikh et al., 2015). Postoperatively, patients are at risk for delayed emergence, hypotonia, seizures, and metabolic acidosis (Brody, 2022). Serious and, in rare cases, fatal perioperative complications have been described in patients with mitochondrial disease undergoing anesthesia, primarily in individual case reports and small series. These reports highlight potential vulnerability rather than establishing definitive causal relationships (Table 4). Representative clinical case reports and institutional alerts describing serious or fatal perioperative events following anesthesia in patients with mitochondrial disease are summarized in Table 4, underscoring the heterogeneity of anesthetic responses and the need for individualized perioperative risk assessment. Recent case reports in pediatric patients with mitochondrial disorders have described acute neurological deterioration temporally associated with perioperative anesthetic exposure, including hypotonia, apnea, and hospitalization (Lopes et al., 2018; Alkuwaiti et al., 2025). Collectively, these cases underscore the need for heightened perioperative vigilance and individualized anesthetic planning, while also illustrating the limited and heterogeneous nature of the available clinical evidence.

**TABLE 4:** Clinical case reports and institutional alerts describing serious or fatal perioperative events following anesthesia in individuals with mitochondrial disease.

Case	Patient	Anesthetic agent	Outcome	References
1	Adult with POLG-related MD	Balanced anesthesia with intravenous agents (e.g., propofol, opioids, muscle relaxants) combined with inhalational agents (volatile anesthetics such as sevoflurane/isoflurane)	Cardiac arrest intraoperatively	Kurnutala and Hubbard (2023), Niezgoda and Morgan (2013)
2	Child with OXPHOS deficiency type 3 (this case report)	Balanced anesthesia with sevoflurane, low-dose propofol, fentanyl, atracurium	Uneventful perioperative course, no metabolic decompensation	Manickam et al. (2024)
3	Child with deficiency in the activity of Complexes I, III, and IV of OXPHOS	Induction: thiopental + fentanyl; maintenance: isoflurane + N_2_O	Agitation, motor focality, degeneration of white matter. Death 5 weeks after surgery	Casta et al. (1997)
4	Teenager with MELAS syndrome	Propofol and rocuronium	Hypothermia and risk of postoperative ventilatory failure	Bolton et al. (2003)
5	Pediatric patient with mitochondrial respiratory chain disorder	Sevoflurane (inhalational)	Intraoperative hypersensitivity to volatile anesthetic → abnormal hemodynamic instability, highlighting mitochondrial vulnerability	Hsieh et al. (2021)
6	Adult with genetically confirmed mitochondrial disease (non-syndromic)	Balanced general anesthesia with volatile agent + opioids	Severe intraoperative cardiovascular instability (arrhythmias, hypotension) requiring pharmacological resuscitation. Postoperative metabolic decompensation	Harbell et al. (2021)
7*	Venezuelan pediatric patients in Chile (series under investigation)	General anesthesia with widely used techniques (volatile agents ± IV)	Severe neurological complications; 4 deaths reported; etiology under investigation (possible mitochondrial vulnerability)	Sociedad de Anestesiología de Chile (SACH) communiqué; Instituto de Salud Pública de Chile (ISP) alert

MD: Mitochondrial disease. MELAS: mitochondrial encephalomyopathy, Lactic Acidosis, and Stroke-like episodes, PRIS: propofol infusion syndrome, POLG: Polymerase Gamma (gene involved in mitochondrial DNA, replication), mtDNA: Mitochondrial DNA, IV: Intravenous.* *“This row summarizes an institutional alert/series (not a peer-reviewed case report). Details are under investigation.”*

## 6 Anesthetic vulnerability in mitochondrial dysfunction: evidence-graded overview

Mitochondrial disorders—including pathogenic mtDNA variants, OXPHOS deficiencies, and secondary mitochondrial dysfunction—are associated with increased perioperative risk due to limited metabolic reserve, impaired redox buffering, and vulnerability to anesthetic-induced inhibition of respiratory chain complexes (Salehpoor et al., 2023). Because the literature includes both peer-reviewed reports and institutional safety alerts, we explicitly separate the evidence below to avoid overstating certainty.

### 6.1 Peer-reviewed evidence (*Clinical case reports, case series, mechanistic studies)*

#### 6.1.1 Volatile anesthetics

- Multiple case reports and biochemical studies show that sevoflurane, isoflurane, and desflurane inhibit Complexes I and II *in vitro* and reduce mitochondrial membrane potential in susceptible tissues (Bains et al., 2006).
- Clinical cases describe postoperative metabolic acidosis, rhabdomyolysis, and respiratory depression in patients with primary mitochondrial disease (Niezgoda and Morgan, 2013).

#### 6.1.2 Propofol

- Propofol inhibits Complexes I and IV and uncouples OXPHOS in isolated mitochondria (Finsterer and Frank, 2016).
- Propofol Infusion Syndrome (PRIS)—characterized by lactic acidosis, rhabdomyolysis, and cardiovascular collapse—has been reported more frequently in settings of underlying mitochondrial stress (critical illness, long infusion times, high doses) (Vanlander et al., 2012).
- Case reports of PRIS-like presentations exist in pediatric patients later confirmed to carry mitochondrial disorders (Shimizu et al., 2020).

#### 6.1.3 Succinylcholine

- Well-documented risk of hyperkalemia and rhabdomyolysis, especially in patients with myopathic or mitochondrial phenotypes (Niezgoda and Morgan, 2013).
- Contraindicated in this patient population based on consistent clinical evidence (Niezgoda and Morgan, 2013).

#### 6.1.4 Opioids, Benzodiazepines, dexmedetomidine

- Generally considered low mitochondrial burden (Hsieh et al., 2017).
- Dexmedetomidine has emerging evidence (preclinical + small clinical series) suggesting protection against mitochondrial swelling and oxidative stress (Lin et al., 2023).

### 6.2 Institutional alerts and non–peer-reviewed signals (preliminary, not Confirmatory)

In Chile, institutional safety communications have recently signaled potential perioperative vulnerabilities in certain pediatric populations. In July 2025, the *Sociedad de Anestesiología de Chile (SACH)* issued a formal report describing five pediatric cases in which previously healthy children subjected to standard anesthetic regimens including sevoflurane, propofol, and fentanyl failed to regain consciousness or exhibited severe neurologic compromise postoperatively, with four resulting in death. These cases occurred without identifiable intraoperative complications such as hypoxia or anaphylaxis, and were managed according to established clinical protocols, prompting internal review and referral to national health authorities for further investigation of underlying risk factors.

### 6.3 Evidence-graded practical recommendations (based on peer-reviewed evidence and institutional alerts)

- Total intravenous anesthesia (TIVA) with carefully titrated propofol (avoiding prolonged or high-dose infusions) (Brody, 2022).
- Adjuncts: dexmedetomidine, short-acting opioids (remifentanil), midazolam (Vanlander et al., 2012).
- Trigger avoidance: strict avoidance of succinylcholine (Niezgoda and Morgan, 2013).
- Cautious use of volatiles, minimizing dose and exposure duration (Brody, 2022).

### 6.4 Perioperative metabolic precautions

- Avoid prolonged fasting (provide glucose-containing IV fluids if needed) (Parikh et al., 2017).
- Monitor: lactate, acid–base status, CK, glucose, and temperature (Parikh et al., 2015).
- Maintain normothermia and adequate oxygen delivery (Parikh et al., 2015).
- Early recognition of postoperative metabolic decompensation (Parikh et al., 2015).

## 7 Mitochondria-targeted therapies

Several synthetic and naturally derived compounds exhibit neuroprotective effects, mainly through the preservation of mitochondrial membrane potential, enhancement of ATP production, reduction of reactive oxygen species (ROS), and modulation of signaling pathways related to apoptosis and inflammation. We propose that such compounds could be beneficial not only for the treatment of mood disorders but also to mitigate risks associated with anesthesia.

### 7.1 Elamipretide

Elamipretide protects against anesthesia and surgery-induced mitochondrial dysfunction by improving ATP production, preserving mitochondrial membrane potential, reducing ROS and preventing oxidative stress as well as the opening of the mitochondrial permeability transition pore (Wu et al., 2015; Wu et al., 2017). By targeting mitochondrial dysfunction, elamipretide shows therapeutic potential for preventing postoperative neurocognitive disorders (PND) in aged subjects (Zuo et al., 2020; Zhao et al., 2019; Zhang et al., 2024a). Preclinical studies suggest elamipretide can improve memory and reduce inflammation, and although clinical studies have focused on other mitochondrial diseases, this mitochondrial effects provide a rationale for its potential therapeutic use in mood disorders (Sabbah et al., 2025; Ciubuc-Batcu et al., 2024).

### 7.2 Melatonin

While direct evidence of melatonin preventing major depressive disorder is limited, its role in regulating circadian rhythms and its anti-inflammatory and neuroprotective effects suggest a potential therapeutic role in depression by mitigating neuroinflammation and improving brain health (Cardinali et al., 2013; Won et al., 2021; Melhuish Beaupre et al., 2021). Studies have observed decreased bedtime melatonin levels in patients with major depression, and these levels may negatively correlate with the severity of depressive symptoms (Wang et al., 2022). Despite these findings, the evidence from clinical trials for the direct use of melatonin as a treatment for core depression symptoms is not yet consistent, requiring more research to clarify its role (Shokri-Mashhadi et al., 2023). The activation of mitochondria-dependent apoptotic pathway is important in the early stages of anesthesia-induced developmental neuroapoptosis (Yon et al., 2006). Melatonin-induced neuroprotection is mediated, at least in part, through inhibition of the mitochondria-dependent apoptotic pathway since melatonin caused an upregulation of the anti-apoptotic protein, bcl-X(L), reduction in anesthesia-induced cytochrome c release into the cytoplasm and a decrease in anesthesia-induced activation of caspase-3, an important step in the activation of DNAses and the formation of the apoptotic bodies (Yon et al., 2006). Melatonin has also been explored for potential applications in pediatric anesthesia (Subhadarshini and Taksande, 2024).

### 7.3 Coenzyme Q10

Studies in young rodents demonstrate that CoQ10 can reduce sevoflurane-induced harm by preventing mitochondrial membrane potential loss, increasing ATP production, and protecting synaptic function, thereby offering a potential therapeutic strategy for patients susceptible to anesthetic neurotoxicity (Xu et al., 2017). Findings suggest CoQ10 could be a key component in developing new interventions to prevent or treat cognitive impairment associated with anesthesia and other mitochondrial diseases (Bhagavan and Chopra, 2005; Hargreaves, 2014). Given its roles as an antioxidant and a mitochondrial restorer, CoQ10 holds promise as a supplement to help manage mood disorders. Clinical studies suggest CoQ10 may also reduce depressive symptoms when added to standard treatments for bipolar depression. However, further research is needed to confirm these findings and to establish its effectiveness and safety as a monotherapy (Pradhan et al., 2021; Mehrpooya et al., 2018; Forester et al., 2015).

### 7.4 Dexmedetomidine

Dexmedetomidine is widely used in clinical anesthesia to optimize anesthesia and analgesia effects and reduce intraoperative adverse reactions. A preclinical study shows that dexmedetomidine could alleviate anxiety-like behavior and cognitive impairment in posttraumatic stress disorder model rats. In clinical studies, the perioperative administration of dexmedetomidine had an anxiolytic effect (Yu et al., 2023). Additionally, it ameliorates brain damage in the intracerebral hemorrhage model by suppressing oxidative stress resulting from the deactivation of the PGC-1α pathway (plays an important regulatory role in cellular metabolism and mitochondrial biosynthesis) and mitochondrial dysfunction (Zhang et al., 2024b). Dexmedetomidine administration in the early postpartum period significantly reduced the incidence of a positive postpartum depression screening and maintained a favorable safety profile (Zhou et al., 2024). In clinical trial, a single dose of sublingual dexmedetomidine reduced the severity of agitation in participants with mild to moderate agitation associated with bipolar disorder after self-administering the medication (Preskorn et al., 2022). Regarding mitochondrial effects, dexmedetomidine alleviates oxidative stress and mitochondrial dysfunction associated with diabetic peripheral neuropathy by downregulating miR-34a to regulate the SIRT2/S1PR1 axis (Lin et al., 2023), besides maintaining the mitochondrial fusion/fission balance through the PKC-ɑ/HO-1 signaling pathway (Song et al., 2022) and preserves neuronal function by promoting mitochondrial biogenesis through the AMPK/PGC-1α pathway (Wang et al., 2025). Dexmedetomidine is proposed as a potentially novel antidepressant with multiple mechanisms of action targeting various depression pathophysiological processes. These mechanisms include modulation of the noradrenergic system, regulation of neuroinflammation and oxidative stress, influence on the Brain-Derived Neurotrophic Factor (BDNF) levels, and modulation of neurotransmitter systems, such as glutamate (Al-Mahrouqi et al., 2023).

### 7.5 Luteoloside

Luteoloside prevented sevoflurane-induced cognitive dysfunction in aged rats and may be associated with the regulation of mitochondrial dynamics. Luteoloside diminished sevoflurane-induced mitochondrial membrane potential disruption and ROS overproduction and protected neurons from sevoflurane-induced apoptosis. Neuroprotective activities of luteoloside may be attributed to its mitochondrial protection by the regulation of mitochondrial dynamics (Zhang et al., 2023). In a mouse sleep deprivation (SD) model exhibits an antidepressant effect and increases active stress coping response to SD stress, possibly by modulating the BDNF/TrkB/ERK/CREB signaling pathway (Ryu et al., 2022). Luteolin glucuronide, such as luteoloside, has been reported to be deconjugated to luteolin. Luteolin was shown to alleviate cognitive deficits, anxiety degree and exploring ability in a triple transgenic AD mouse model. Luteolin targeted oxidative stress and mitochondrial dysfunction induced by Aβ, subsequently suppressed neuronal apoptosis (He et al., 2023). Preclinical studies and limited clinical evidence on the antidepressant and neuroprotective effects of luteolin allow for an exploration of its antidepressant potential. It acts as an antidepressant by regulating neurotransmitters, reducing oxidative stress, and calming inflammation (Zhou et al., 2025).

### 7.6 Vitexin

In the few studies that explore the effects of vitexin on the mechanisms implicated in the pathogenesis of neurodegeneration, it has demonstrated great therapeutic potential to be explored in the reduction of oxidative stress and neuroinflammation (Lima et al., 2018). Vitexin has also been suggested to have antidepressant effects, because it exerted antidepressant-like effects by increasing the catecholamine levels in the synaptic cleft as well as through interactions with the serotonergic 5-HT1A, noradrenergic α2, and dopaminergic D1, D2, and D3 receptors (Can et al., 2013). Vitexin showed neuroprotective potential by ameliorating mitochondrial dysfunction, and associated AIM2 inflammasome activation in experimental trauma induced neuropathic pain (Pal et al., 2025). Vitexin inhibited the isoflurane-induced cell cytotoxicity and weakened isoflurane-induced neuroinflammation and oxidative stress pathways in PC12 cells (Chen et al., 2016). Vitexin protects sevoflurane-induced neuronal apoptosis in brain, through HIF-1α, VEGF and p38 MAPK signaling pathway and suppressed Bax protein expression in sevoflurane-induced newborn rat or human neuroglioma cells, which may be assisted adverse reactions during anesthetic in clinical application (Lyu et al., 2018). One study demonstrated that vitexin protected H9c2 cells from ischemia/reperfusion induced mitochondrial dysfunction, significantly reducing ROS levels; improving mitochondrial activity, mitochondrial membrane potential and ATP content; markedly increasing MFN2 expression and reducing the recruitment of Drp1 in mitochondria (Xue et al., 2020). Clinical effects of vitexin on the mechanisms implicated in the pathogenesis of neurodegeneration, it has demonstrated great therapeutic potential to be explored in the reduction of oxidative stress and neuroinflammation.

### 7.7 Phenelzine

Phenelzine, an antidepressant, may offer neuroprotection by mitigating mitochondrial dysfunction, primarily through its role as an aldehyde scavenger that protects mitochondria from lipid peroxidation products like 4-hydroxynonenal (4-HNE) (Cebak et al., 2017; Singh et al., 2013). By reducing oxidative damage to mitochondria, phenelzine can improve their respiratory function and energy production, which is relevant to mood disorders like depression where mitochondrial dysfunction is implicated. This protective mechanism is separate from its well-known function as a monoamine oxidase inhibitor (MAOI), by acting on mitochondrial pathways (Matveychuk et al., 2022; Cebak et al., 2017). While phenelzine has demonstrated mitochondrial protection in injury models, there is limited specific research on its use as a pre-anesthetic. Medicines used for general anesthesia and certain types of local anesthesia can increase the risk of dangerous side effects of phenelzine (Harbell et al., 2021). However, its mechanism of protecting against oxidative damage is a potentially relevant factor to consider.

### 7.8 Pramipexole

Pramipexole demonstrates neuroprotective effects by directly counteracting mitochondrial dysfunction and oxidative stress, often acting as an antioxidant. It works by blocking the mitochondrial permeability transition pore (mtPTP), restoring mitochondrial membrane potential, reducing cytochrome c release, and promoting mitophagy (Andrabi et al., 2019; Tang et al., 2021). Early exposure to general anesthesia can cause mitochondrial damage and cognitive deficits. Pramipexole provides long-lasting protection against cognitive impairments observed when very young animals are exposed to anesthesia during peak brain development, demonstrating the ability to prevent such deficits by preserving mitochondrial function (Boscolo et al., 2013). The D_2_/D_3_ receptor agonist pramipexole has clinical efficacy as an antidepressant, but its neural mechanisms are unknown (Mah et al., 2011). The connection between pramipexole, mitochondrial function, and depression suggests that targeting mitochondrial health could be a promising strategy for developing new and more personalized antidepressant treatments (Li et al., 2024b; Allen et al., 2018; Wei et al., 2026).

Given the metabolic vulnerability of patients with MD, anesthetic management should prioritize minimizing mitochondrial toxicity and reducing catabolic stress.

**FIGURE 4:**
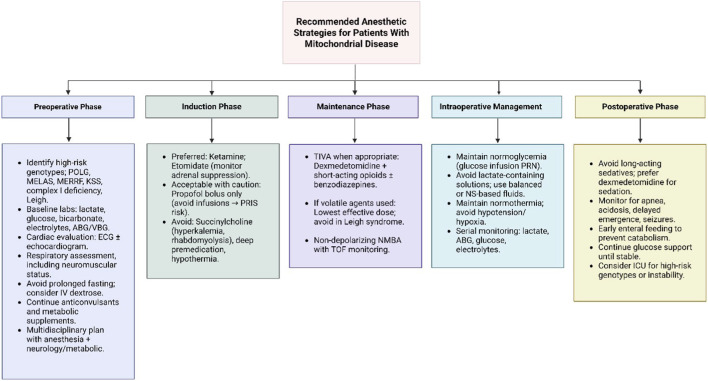
Perioperative anesthetic management protocol for mitochondrial disease. This flowchart provides a structured overview of recommended strategies to reduce perioperative metabolic stress and anesthetic-related risk in patients with mitochondrial disorders. The guidance is derived from published case series, expert consensus, and mechanistic data on anesthetic–mitochondrial interactions. It is not a standardized clinical guideline and should be adapted to individual patient phenotype and genotype (Niezgoda and Morgan, 2013; Brody, 2022; Morgan et al., 2002; Falk, 2010; Alkuwaiti et al., 2025).

Although no anesthetic regimen can be guaranteed safe for all MD phenotypes, certain practices are commonly regarded as lower-risk based on current evidence (Figure 4). Figure 4 presents a structured protocol for perioperative management in patients with mitochondrial disorders. It integrates evidence from case reports, expert consensus, and mechanistic studies to guide strategies that reduce metabolic stress and anesthetic-related risks. Key considerations include minimizing fasting, ensuring glucose availability, careful selection and titration of anesthetic agents, maintaining normothermia, and monitoring bioenergetic endpoints such as lactate, redox balance, and metabolic flexibility.

Mitochondrial dysfunction underlies both mood disorder vulnerability and perioperative anesthetic risk, yet these domains are rarely analyzed together. This section outlines the shared bioenergetic, redox, calcium, and circadian mechanisms that unify both fields, identifies overlapping biomarkers, and highlights the clinical implications for risk stratification and anesthetic management.

## 8 Bridging mood disorders and anesthetic vulnerability: shared mitochondrial stress pathways

Growing evidence indicates that the mitochondrial pathways implicated in mood and circadian dysregulation overlap substantially with those that determine anesthetic tolerance and perioperative metabolic stability (Stein and Imai, 2012; Azouaoui et al., 2023; Greenwood et al., 2024; Brody, 2022). This convergence provides the mechanistic bridge linking the two domains and clarifies why individuals with impaired mitochondrial function—whether clinically manifest or subclinical—may be vulnerable both to mood disturbances and to anesthetic-related complications.

- Preoperative metabolic assessment (when mitochondrial vulnerability is suspected):
  – Lactate: elevated values (commonly > 2 mmol/L relative to normal resting physiology) suggest limited oxidative metabolism (Greenwood et al., 2024).
  – NAD^+^/NADH ratio: Low NAD^+^/NADH ratios relative to typical physiological ranges (e.g., ratios below ∼5–10 in plasma or select tissues, reflecting a more reduced redox state and decreased metabolic flexibility) may indicate impaired redox balance under metabolic stress conditions. Interpretation should be context-specific, as values differ by tissue type and method (Stein and Imai, 2012; Azouaoui et al., 2023).
- Oxidative stress markers: Increased oxidative damage biomarkers may indicate oxidative stress. For example, plasma 8-OHdG levels above ∼0.5–1.0 ng/mL (compared with typical healthy ranges) or elevated lipid peroxidation products such as malondialdehyde (∼1–5 µM) or F2-isoprostanes (∼20–60 pg/mL) have been associated with oxidative damage in clinical and experimental studies (Graille et al., 2020; Wu et al., 2004; Nielsen et al., 1997; van’t Erve et al., 2017).
- mtDNA measures: Reduced mtDNA copy number relative to typical adult ranges (∼100–600 copies per cell in peripheral blood) or the presence of mtDNA variants known to impair OXPHOS may indicate mitochondrial dysfunction or vulnerability. Interpretation should be context-dependent, considering tissue type, age, and analytical method (Mohamed Yusoff et al., 2025; Xia et al., 2017).
- Shared pathophysiological mechanisms (mood–anesthesia interface):
  – Reduced OXPHOS reserve → increased sensitivity to hypoxia, cold, and fasting stress (Casanova et al., 2023).
  – Disrupted Ca^2+^ handling → increased vulnerability to agents affecting Complexes I and III (Michelucci et al., 2021).
  – Elevated baseline ROS → lower threshold for perioperative metabolic toxicity (Zhang et al., 2024b).
  – Low metabolic flexibility → poor tolerance of anesthetic and postoperative stress (Qiao et al., 2023).
- Perioperative management considerations:
  – Avoid prolonged fasting; consider IV glucose-containing fluids when appropriate (Parikh et al., 2017).
  – Minimize volatile anesthetics in patients with suspected Complex I dysfunction (Brody, 2022).
  – Avoid prolonged propofol infusions in those at risk for Complex I impairment (Parikh et al., 2017).
  – Maintain normothermia to reduce metabolic load (Manickam et al., 2024).
  – Consider carefully titrated TIVA when clinically justified (Brody, 2022).
- Translational and research implications:
  – Define bioenergetic endpoints for future trials (lactate, redox balance, temperature, cognitive–affective recovery) (Caddye et al., 2023).
  – Develop shared risk biomarkers applicable to psychiatric and perioperative populations (Nunes et al., 2025).
  – Integrate mitochondrial profiling into preoperative risk-stratification frameworks (Yang et al., 2024).

## 9 Conclusion

In conclusion, mitochondrial dysfunction represents a promising yet complex target in the pathophysiology of mood disorders. Although mitochondria-targeted strategies are appealing, their clinical translation must be pursued with caution, given the multifactorial nature of depression and bipolar disorder. Future research should focus on distinguishing primary mitochondrial defects from downstream consequences and on developing personalized interventions guided by biomarkers that capture mitochondrial bioenergetics and resilience in the human brain.
